# QUALITY CARE AND CONFLICTING RIGHTS AMONG RESIDENTS WITH DEMENTIA, FAMILIES, AND STAFF IN LONG-TERM CARE FACILITIES

**DOI:** 10.1093/geroni/igac059.1759

**Published:** 2022-12-20

**Authors:** Angela Perone

**Affiliations:** University of California - Berkeley, Berkeley, California, United States

## Abstract

Conflicting rights between residents with dementia and staff pose unique problems for quality of care in long-term care (LTC) facilities. Residents relying on staff for care may be afraid to express concerns. A disproportionate female (and often women of color) staff may be exposed to discrimination that stems from a resident’s loss of executive functioning and inability to control emotions. Family often find themselves somewhere in the middle. This study, thus, poses two research questions: (1) How do residents, families, and LTC staff understand their rights when conflicts arise between residents with dementia and staff?; (2) How can we leverage collective expertise of residents, families, and LTC staff to deliver a more holistic approach for addressing these conflicts? To answer these questions, I employed a multi-method qualitative design using semi-structured interviews (n=90) and participant observation (n=8 months). Building on legal consciousness theory—which explains how individuals invoke (or do not invoke) legal principles to define everyday experiences—findings revealed that staff rarely invoked legal framings to describe interactions whereas residents readily invoked rights rhetoric. Families mostly avoided rights rhetoric and instead focused on storytelling. All groups (residents, staff, and families) employed an array of emotional literacy and life experiences to navigate conflicts that coalesced around empathy. This paper concludes with research, policy, and practice suggestions for invoking principles of empathy to advance quality of care for residents with dementia by improving interactions among residents, staff, and family and workforce conditions for staff that flow from these interactions.

